# Fighting the storm: could novel anti-TNFα and anti-IL-6 *C. sativa* cultivars tame cytokine storm in COVID-19?

**DOI:** 10.18632/aging.202500

**Published:** 2021-01-19

**Authors:** Anna Kovalchuk, Bo Wang, Dongping Li, Rocio Rodriguez-Juarez, Slava Ilnytskyy, Igor Kovalchuk, Olga Kovalchuk

**Affiliations:** 1 Pathway Research Inc., Lethbridge, AB T1K7X8, Canada; 2 University of Calgary, Cumming School of Medicine, Calgary, AB T2N 1N4, Canada; 3 University of Lethbridge, Lethbridge, AB T1K3M4, Canada

**Keywords:** COVID-19, SARS-CoV2, cytokine storm, TNFα, IL-6, fibrosis, medical cannabis

## Abstract

The main aspects of severe COVID-19 disease pathogenesis include hyper-induction of proinflammatory cytokines, also known as ‘cytokine storm’, that precedes acute respiratory distress syndrome (ARDS) and often leads to death. COVID-19 patients often suffer from lung fibrosis, a serious and untreatable condition. There remains no effective treatment for these complications. Out of all cytokines, TNFα and IL-6 play crucial roles in cytokine storm pathogenesis and are likely responsible for the escalation in disease severity. These cytokines also partake in the molecular pathogenesis of fibrosis. Therefore, new approaches are urgently needed, that can efficiently and swiftly downregulate TNFα, IL-6, and the inflammatory cytokine cascade, in order to curb inflammation and prevent fibrosis, and lead to disease remission.

*Cannabis sativa* has been proposed to modulate gene expression and inflammation and is under investigation for several potential therapeutic applications against autoinflammatory diseases and cancer. Here, we hypothesized that the extracts of novel *C. sativa* cultivars may be used to downregulate the expression of pro-inflammatory cytokines and pathways involved in inflammation and fibrosis.

Initially, to analyze the anti-inflammatory effects of novel *C. sativa* cultivars, we used a well-established full thickness human 3D skin artificial EpiDermFTTM tissue model, whereby tissues were exposed to UV to induce inflammation and then treated with extracts of seven new cannabis cultivars. We noted that out of seven studied extracts of novel *C. sativa* cultivars, three (#4, #8 and #14) were the most effective, causing profound and concerted down-regulation of COX2, TNFα, IL-6, CCL2, and other cytokines and pathways related to inflammation and fibrosis. These data were further confirmed in the WI-38 lung fibroblast cell line model. Most importantly, one of the tested extracts had no effect at all, and one exerted effect that may be deleterious, signifying that careful cannabis cultivar selection must be based on thorough pre-clinical studies.

The observed pronounced inhibition of TNFα and IL-6 is the most important finding, because these molecules are currently considered to be the main targets in COVID-19 cytokine storm and ARDS pathogenesis.

Novel anti-TNFα and anti-IL-6 cannabis extracts can be useful additions to the current anti-inflammatory regimens to treat COVID-19, as well as various rheumatological diseases and conditions, and ‘inflammaging’ - the inflammatory underpinning of aging and frailty.

## INTRODUCTION

To date, raging pandemic of COVID-19 disease caused by the SARS-CoV2 virus has affected over 80 million people and claimed over 1,750,000 lives worldwide. SARS-CoV2 has human-human transmission and spreads easily via airborne and contact routes; its R_0_ is currently estimated to be 2-2.5 [1]. COVID-19 has a rather broad spectrum of clinical manifestations, ranging from asymptomatic, to mild flu-like disease, to pneumonia, that in some cases can further progress to acute respiratory distress syndrome (ARDS), major organ failure and death. Approximately 20% of COVID-19 cases are serious or severe, and death rate is currently estimated to be around 10%. While elderly and individuals with pre-existing conditions are among the most affected, it has recently become apparent that COVID-19 affects all age groups.

Along with virus levels, the key aspects of the severe COVID-19 disease pathogenesis include increasing hyper-induction of proinflammatory cytokines, which is also known as ‘cytokine storm' that precedes acute respiratory distress syndrome (ARDS) [2, 3]. It is now well-established that the severity of COVID-19 is due to the host immune response [4] and that the cytokine storm, a host-mediated response, is a key feature of immunopathogenesis of COVID-19 infection [4, 5].

Overall, various plasma cytokines and chemokines were reported to be deregulated in COVID-19 patients; these include TNF-α, interleukins (IL-1, IL-2, IL- 4, IL-7, IL-10, IL-12, IL-13, IL-17), macrophage colony-stimulating factor (MCSF), IP-10, MCP-1 (C-C motif chemokine 2, CCL2), MIP-1α, hepatocyte growth factor (HGF), IFN-γ, CCL3, CCL5 and many others [6]. Cytokine levels correlate with disease severity [7]. Patients with moderate COVID-19 disease had elevated levels of TNFα and IL-6, and in severe COVID-19 cases the production of IL-6 and TNF-α and other cytokines was profoundly increased [7]. Moreover, patients requiring ICU admission had higher levels of IL-6, IL-2, IL-7, IL-10, GCSF, IP10, CCL2, MIP1A, and TNFα than did those not requiring ICU admission, suggesting that the cytokine storm was important in COVID-19 pathogenesis [8, 9].

Of the cytokine milieu, TNFα and IL-6 play key roles in cytokine storm and are likely to be responsible for the escalation in disease severity [10–12]. TNFα is an inflammatory cytokine that stimulates and maintains cellular activation and migration of leukocytes to inflammatory sites. TNF acts by binding to its receptors (TNFR) that are located throughout the body. Interaction of TNF with receptors causes increased expression of other cytokines (IL-1 and IL-6) and chemokines, which, in turn, activate leukocytes, suppresses regulatory T cells, causes production of MMP proteins which degrade tissues and induce apoptosis [13]. IL-6 is another important player in the acute host response to infection whereby it promotes inflammation, immune reactions, and hematopoiesis. Long-term elevation of IL-6 levels maintains chronic inflammation and autoimmunity, making IL-6 one of the main druggable targets in autoinflammatory and autoimmune disorders [14].

Even though TNFα- and IL-6-mediated cytokine storm and ARDS have been previously well-documented in SARS, MERS, as well as in severe cases of influenza [3, 15], there still is no effective treatment for this grievous complication. Therefore, new approaches are urgently needed that can efficiently and swiftly block TNFα, IL-6 and inflammatory cytokine cascades and thus curb inflammation and lead to disease remission.

Furthermore, COVID-19 convalescents face a long recovery and may be at risk of developing pulmonary fibrosis (PF), a debilitating complication that is very hard to treat [16]. Mechanisms of PF are not fully understood, albeit it has been established that inflammatory cytokines and chemokines, such as IL-1, IL-6, TNFα, C-C motif chemokines are important in its etiology [5, 17]. New therapies are much needed to prevent and mitigate pulmonary fibrosis complications in COVID-19 patients. Since COVID-19, and especially ARDS patients are extremely weak and vulnerable, it would be crucial that novel anti-cytokine storm and anti-fibrosis therapies have minimal side effects.

*Cannabis sativa* has been proposed to modulate gene expression and inflammation and is under investigation for several potential therapeutic applications against autoinflammatory diseases and cancer. Therefore, we hypothesized that extracts of novel *C. sativa* cultivars may be used to downregulate expression of pro-inflammatory cytokines and pathways involved in inflammation and fibrosis.

## RESULTS

### Cannabis extracts affect the expression of inflammation-related genes and proteins in the EpiDermFT model

For the initial analysis of the anti-inflammatory effects of novel *C. sativa* cultivars, we used a well-established full thickness human 3D skin artificial EpiDermFT^TM^ tissue model, whereby tissues were exposed to UV to induce inflammation and then treated with extracts of seven new cannabis cultivars. Upon original screening of over 200 extracts, seven extracts of cultivars #4, #6, #8, #12, #13, #14, #15, were identified for further analysis.

**Figure 1:**
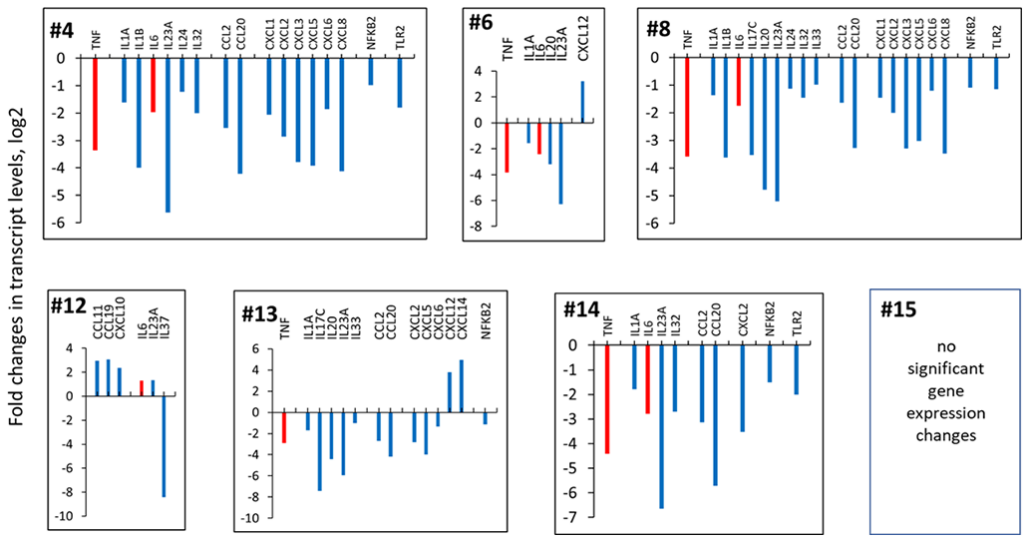
**Effects of novel *C. sativa* extracts on the levels of pro-inflammatory cytokines.** To induce inflammation, human 3D EpiDermFT tissues were exposed to UV. Upon exposure, tissues were incubated with extracts or vehicle (DMSO) for 24 h. Three tissues were used for each condition. The differences between all experimental groups were examined using the likelihood ratio test (LRT) test implemented in DESeq2. The reduced model included the intercept and the full model was the experimental group (Cannabis extracts and controls). Multiple comparisons adjustment of p-values was done using Benjamini-Hochberg procedure [63]. Specific comparisons between groups were done using *results()* function with *contrast* argument specified. Genes with adjusted p-values below 0.05 were considered significant. Data are shown as log 2 fold changes as compared to UV-induced tissues. All changes shown here are statistically significant, p adj <0.05, ANOVA-like analysis and pair-wise comparison.

Analysis of global gene expression profiling revealed that 5 new extracts strongly down-regulated expression of interleukins, pro-inflammatory cytokines, C-C motif chemokines and C–X–C subfamily cytokines involved in ADRS and other autoinflammatory conditions (padj<0.05) (Figure 1 and Table 1).

**Table 1:** Effects of cannabis cultivars on the levels inflammation and fibrosis-related genes human 3D EpiDermFT tissues, as studied by the global transcriptome profiling using RNAseq.

**LINE**	**#4**	**#8**	**#14**	**#13**	**#6**	**#12**	**#15**
**Inflammation- and fibrosis-related genes**							
**TNF**	**-3.4**	**-3.6**	**-4.4**	**-2.9**	**-3.9**		
IL1A	-1.6	-1.4	-1.8	-1.7	-1.6		
IL1B	-4.0	-3.6			-2.4		
**IL6**	**-2.0**	**-1.8**	**-2.8**		**-2.4**	**1.3**	
IL17C		-3.5		-7.4			
IL20		-4.8		-4.4	-3.2		
IL23A	-5.6	-5.2	-6.7	-6.0	-6.3	1.3	
IL24	-1.2	-1.1					
IL32	-2.0	-1.5	-2.7		3.2		
IL33		-1.0		-1.0			
IL37						-8.4	
CCL2	-2.5	-1.6	-3.1	-2.7			
CCL20	-4.2	-3.3	-5.7	-4.2			
CXCL1	-2.1	-1.4					
CXCL2	-2.9	-2.0	-3.5	-2.8			
CXCL3	-3.8	-3.3					
CXCL5	-3.9	-3.0		-4.0			
CXCL6	-1.8	-1.2		-1.3			
CXCL8	-4.1	-3.5					
CXCL10						2.3	
CXCL12				3.8	3.2		
CXCL14				5.0			
NFKB2	-1.0	-1.1	-1.5	-1.1			
PTGS2	-3.3	-2.5	-3.7	-2.9	-3.3	1.6	
TLR2	-1.8	-1.2	-2.0				
**Fibrosis-related genes**							
MMP1	-2.7	-1.8					
MMP3		-1.8					
MMP7		2.7		3.6	2.8		
MMP8		-1.5		-2.0			
MMP10	-1.7	-1.7	-1.5				
MMP11				3.2	2.9		
MMP19		-1.0		-1.1			
WNT2	-2.2	-1.5	-2.1	-1.5	-2.2		
WNT5A	-1.5	-1.2	-1.5	-1.4	-1.3		
FZD4	-1.2						
ICAM1	-1.5	-1.4	-2.2		-1.8		
ICAM5	-1.6	-2.0					

Data are shown as log 2 fold changes as compared to UV-induced tissues. All changes shown here are statistically significant, p adj <0.05, ANOVA-like analysis and pair-wise comparison.

*TNFα and IL-6*: Application of the extracts # 4, #6, #8 and #14 down-regulated both TNFα and IL-6. Extract #13 downregulated TNFα but not IL-6. Interestingly, extract #12 upregulated the expression of IL-6 and IL-23A, pro-inflammatory chemokines, and down-regulated the expression of anti-inflammatory IL-37. Application of extract#15 did not result in any statistically significant gene expression changes (Figure 1 and Table 1).

**Figure 2:**
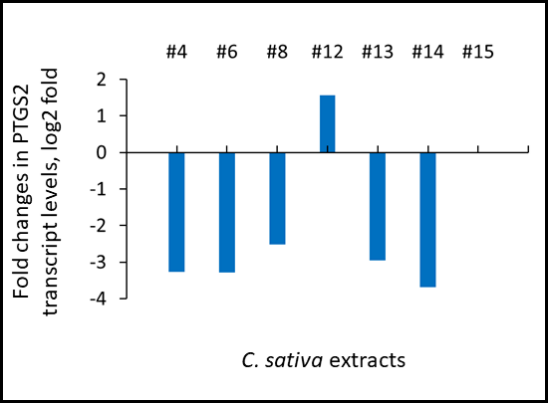
**Effects of novel *C. sativa* extracts on the levels of PTGS2 gene expression as studied by the global transcriptome profiling using RNAseq.** Induction of inflammation and treatments were described in the legend to Figure 1. Data are shown as log 2 fold changes as compared to UV-induced tissues. All changes shown here are statistically significant, p adj <0.05, ANOVA-like analysis and pair-wise comparison.

*COX2:* Moreover, extracts #4, #6, #8, #13 and #14 significantly down-regulated the expression of prostaglandin-endoperoxide synthase 2 (PTGS2) gene that encodes for cyclooxygenase 2 (COX2). Extract #15 had no effects on PTGS2 levels, whereas extract #12 caused an upregulation of PTGS2 expression (Figure 2).

**Figure 3:**
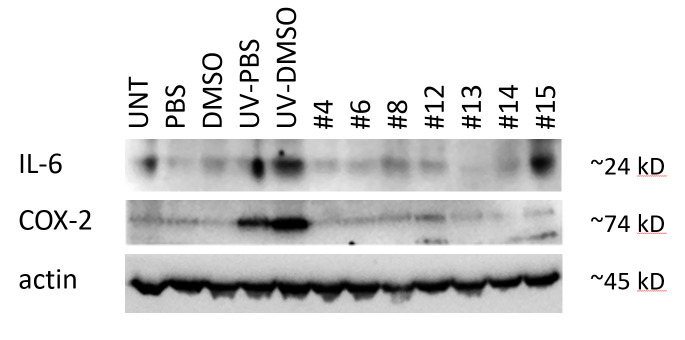
**Effects of novel *C. sativa* extracts on the levels of IL-6 and COX2 in human EpiDerm FT tissues.** To induce inflammation, tissues were exposed to UV. Upon exposure, tissues were incubated with extracts or vehicle (DMSO) for 24 h. Three tissues were used for each condition. Western blot analysis was performed using antibodies against IL-6 and COX2 as detailed in the Methods section. “UNT” – untreated tissues; “PBS” - 15 μl of 30% glycerol in PBS was applied to the tissues and no exposure was done; “DMSO” - 15 μl of DMSO (0.017% in 30% glycerol-PBS) was applied to the tissues and no exposure was done; “UV-PBS” - tissues were exposed to UV and 15 μl of 30% glycerol-PBS was applied to them; “UV-DMSO” – tissues were exposed to UV and 15 μl of DMSO (0.017% in 30% glycerol-PBS) was applied to them; “#4” through “#15” - tissues were exposed to UV and 15 μl of extracts in DMSO was applied to them.

We further explored the effects of *C. sativa* extracts on the levels of IL-6 and COX2 proteins using western immunoblotting, and found that all extracts, except #15, downregulated UV-induced IL-6 expression and all extracts downregulated UV-triggered COX2 induction (Figure 3). Interestingly, application of extract #12 downregulated IL-6 on the protein level, but not on the level of the transcript. This is an interesting finding that may suggest the presence of post-transcriptional regulation of IL-6 expression via small interfering RNAs and the potential effects of cannabis extracts on these processes.

*IL-1, IL-17, IL-23*: Along with two key regulators of cytokine storm – TNFα and IL-6, *C. sativa* extracts also affected the levels of other key pro-inflammatory interleukins – IL-1, IL-17, IL-23 (Figure 1 and Table 1). Here, we found that extracts #4 and #8 downregulated both IL-1α and IL-1β (Figure 1 and Table 1). Further, extracts #4, #6, #8, #13 and #14 downregulated, while extract #12 upregulated IL-23A, a member of the IL-12 family of cytokines with pro-inflammatory properties [18]. Extracts #8 and #13 downregulated IL-17C, a pro-inflammatory cytokine and a member of IL-17 family, that, together with IL-23 mediates inflammation in psoriasis, psoriatic arthritis, and ankylosing spondylitis [19].

*TLR:* Three extracts, #4, #8 and #14 downregulated the levels of Toll-like receptor 2 (TLR2), which has been implicated in numerous inflammatory diseases [20], including pulmonary diseases and ARDS [21].

*NFKB2:* In addition, extracts #4, #8, #13 and #14 significantly down-regulated the expression of NFKB2 gene, which has been often referred to as a prototypical proinflammatory signaling pathway. NF-κB is usually upregulated by IL-1 and TNFα, and play important roles in the expression of other proinflammatory genes [22].

### Cannabis extracts affect the levels of fibrosis-related genes in the EpiDermFT model

We next looked at the effect of cannabis extracts on the levels of fibrosis-related mRNAs. Global gene expression profiling analysis revealed that extracts #4, #8, #13 and #14 downregulated CCL2, also known as MCP-1 (Figure 1 and Table 1), which is an important hallmark of fibrosis, and has been indicated as a potential druggable anti-fibrotic target [23]. Several extracts down-regulated MMPs (Table 1).

Extracts #4, #6, #8, #14 and #13 also down-regulated WNT2 and WNT5a. WNT signaling alterations have been linked to pathogenesis of a variety of diseases and conditions, including pulmonary fibrosis [24, 25]. Furthermore, extracts also affected the levels of iCAM1 and iCAM5 genes.

One more important pro-fibrotic protein is CXCL12, and its down-regulation was shown to dampen fibrocyte recruitment and collagen deposition [26]. In our study, extracts #6 and #13, along with down-regulation of numerous pro-inflammatory cytokines, upregulated CXCL12.

### In-depth analysis reveals pathways affected by cannabis extracts in EpiDermFT tissues

Having seen cannabis extract-induced changes in pro-inflammatory and pro-fibrotic genes, we further conducted an in-depth analysis of the effects of the extracts on global signalome using Pathview Bioconductor platform. We found that extracts # 4, #8, #14 significantly down-regulated cytokine-cytokine receptor interaction pathway, rheumatoid arthritis pathway, chemokine signalling, Toll-like receptor signalling, JAK-STAT signalling and other pathways involved in inflammation, immunity and autoimmunity, as well as tissue remodeling and fibrosis. Contrarily, extract #12 upregulated these pathways (Table 2 and Supplementary Figure 1).

**Table 2:** Pathways most significantly deregulated in EFT-400 tissues upon treatments with extracts of new *C. sativa* cultivars #4. #14, #8 and #12.

**KEGGID**	**Pvalue**	**Term**	**Genes**
**Cultivar #4**
**DOWNREGULATED PATHWAYS**			
5323	1.79E-11	Rheumatoid arthritis	6374;1437;6364;51561;3553;2919;6347;4312;6372;3569;3576;2321;3383;7097;3552;3689;7124
4060	4.22E-11	Cytokine-cytokine receptor interaction	6374;1437;6364;51561;2921;3553;2919;3976;57007;2920;6347;6372;3569;3575;7850;3576;2321;3552;7133;3082;7124;84957;1440;11009;23529
4514	2.51E-06	Cell adhesion molecules (CAMs)	25945;4897;80380;3383;3696;214;3689;1364;257194;1366;29126;23562;3134
4062	3.82E-05	Chemokine signaling pathway	6374;6364;2921;2919;2920;6347;6372;3055;2791;3576;4792;5908;57580;5604;114
4630	0.000634346	Jak-STAT signaling pathway	3598;1437;51561;3976;3569;3575;6775;81848;1440;11009;23529
4210	0.000993993	Apoptosis	3553;330;3656;3552;4792;11213;5533;637;7124
4620	0.008055248	Toll-like receptor signaling pathway	3553;3569;3576;7097;4792;5604;7124
4660	0.019535451	T cell receptor signaling pathway	1437;4792;5533;4773;5604;7124;4794
**Cultivar #14**			
**DOWNREGULATED PATHWAYS**			
4621	5.24E-06	NOD-like receptor signaling pathway	6347;2920;3569;7128;330;8767;7124;4792
4060	9.06E-06	Cytokine-cytokine receptor interaction	51561;6347;3976;2920;57007;3569;6364;7124;7133;23529;7850;84957;3552
5323	4.01E-05	Rheumatoid arthritis	51561;6347;3383;3569;6364;7124;3552;7097
4514	0.000993116	Cell adhesion molecules (CAMs)	3383;80380;25945;214;4897;1364;257194
4620	0.010832243	Toll-like receptor signaling pathway	3569;7124;4792;5606;7097
4062	0.04389252	Chemokine signaling pathway	6347;2920;3055;2791;6364;4792
4210	0.003683526	Apoptosis	330;7124;4792;3656;5533;3552
**Cultivar #8**			
**DOWNREGULATED PATHWAYS**			
4060	1.45E-13	Cytokine-cytokine receptor interaction	1440;6364;2921;3976;3553;51561;6374;2920;3589;57007;650;2919;6347;1437;3624;3576;3569;50604;3552;6372;3082;7124;51330;7133;84957;11009;23529
5323	7.81E-12	Rheumatoid arthritis	6364;3553;51561;6374;3589;4312;2919;6347;1437;3576;3569;3552;6372;3383;7097;7124;3689
4630	9.93E-05	Jak-STAT signaling pathway	1440;3976;51561;3589;3598;1437;3569;50604;81848;6775;11009;23529
4514	0.000889035	Cell adhesion molecules (CAMs)	25945;23562;4897;80380;3383;257194;3696;3689;3134
4062	0.000939189	Chemokine signaling pathway	6364;2921;6374;2920;2919;6347;2791;3576;6372;4792;9564;57580
4620	0.005637004	Toll-like receptor signaling pathway	3553;3576;3569;4792;7097;148022;7124
4210	0.010118422	Apoptosis	3553;3656;3552;4792;7124;330;5533
**Cultivar #12**			
**UPREGULATED PATHWAYS**			
4060	0.000239246	Cytokine-cytokine receptor interaction	3976;6376;6356;650;4982;7852;3627;6363;8995;3569;51561
4010	0.003164277	MAPK signaling pathway	1850;2353;1843;3164;2872;3725;3727;11221;1647;3303;22808
5323	0.008471825	Rheumatoid arthritis	2353;3725;5228;3569;51561
4512	0.045361608	ECM-receptor interaction	22801;1311;1301;1281
4510	0.047132189	Focal adhesion	80310;22801;3725;1311;1301;5228;1281
4620	0.047386067	Toll-like receptor signaling pathway	2353;3725;3627;3569

Pathways were generated using KEGG and rendered by Pathview. Details are provided in Materials and Methods.

### Correlation between extract composition and molecular effects

Overall, our study revealed that cannabis extracts exerted different effects on the 3D tissue inflammation model - some profoundly down-regulated pro-inflammatory cytokines and pro-fibrotic molecules, some affected only several key cytokines, some did not cause any significant changes at all (extract #15), while extract #12 promoted expression of pro-inflammatory genes. This is a very important finding that shows that cannabis is non-generic. Indeed, cultivars have unique profiles of cannabinoids and terpenes that can potentiate each other [27], and hence extracts of different cultivars may have different medicinal properties, even though the ratios of major cannabinoids (THC and CBD) may be similar. Hence each *C. sativa* cultivar has to be thoroughly evaluated for its medicinal properties.

To find whether there was any correlation between the level of cannabinoids in the extracts and the efficiency of the extracts in downregulation of inflammation- and fibrosis-related genes, we analyzed the concentration of THC, CBD, CBGA and CBN in flowers and in the extracts (Table 3). We then ranked the efficiency of the extracts by summing up the values for downregulation of all genes in Table 1. Extracts ranked #4, #8, #14, #13, #6, #12, #15, with #4 being the most efficient and #15 the least efficient. We then correlated the concentration of individual cannabinoids with the efficiency of extracts. We found weak positive (0.24) correlation with the level of total THC (THC and THCA) and weak negative correlation with total CBD (CBD and CBD-A), CBGA and CBN, -0.29, -0.32 and -0.32, respectively. We next analyzed the presence and the concentration of terpenes in three extracts, #8, #6 and #12, with #8 being the best (and equal to #4), #6 being an average, and #12 one of the worst. We found that extract #8 was dominant in β-caryophyllene and caryophyllene oxide, while extract #6 was dominant in α-bisabolol and guaiol, and extract #12 in linalool and guaiol. Further studies are needed to establish the roles of terpenes and their effects on inflammation.

**Table 3:** Level of single and total cannabinoids in flowers and extracts of selected *C. sativa* cultivars.

**Flowers**	**Total THC, %**	**Total CBD, %**	**CBGA, %**	**CBN, %**	**TOTAL Cannabinoids**
**#4**	14.7	0.76	0.1	0.06	15.62
**#6**	4.43	9.61	1.5		15.54
**#8**	14.72	0.14	0.22	0.02	15.1
**#12**	20.13	0.59	0.45	0.05	21.22
**#13**	16.49	0.16	0.17	0.03	16.85
**#14**	21.5	1.35	1.02		23.87
**#15**	14.57	0.46	0.1	0.14	15.13
**Extracts**	**Total THC, %**	**Total CBD, %**	**CBGA, %**	**CBN, %**	**TOTAL Cannabinoids**
**#4**	33.6	1.72	0.32	0.14	35.78
**#6**	10.3	23.4	3.4	0.1	37.1
**#8**	32.5	0.33	0.49	0.05	33.37
**#12**	43.2	1.8	0.92	0.12	46.04
**#13**	38.5	1.2	0.39	0.12	40.21
**#14**	44.3	1.1	0.23	0.32	45.63
**#15**	32.5	0.9	0.23	0.35	33.63
**Extracts/molarity, μM**	**THC**	**CBD**	**CBGA**	**CBN**	**TOTAL Cannabinoids**
**#4**	10.69	0.55	0.10	0.05	N/A
**#6**	3.28	7.44	1.07	0.03	N/A
**#8**	10.34	0.10	0.15	0.02	N/A
**#12**	13.74	0.57	0.29	0.04	N/A
**#13**	12.24	0.38	0.12	0.04	N/A
**#14**	14.09	0.35	0.07	0.10	N/A
**#15**	10.34	0.29	0.07	0.11	N/A

### Cannabis extracts inhibit COX-2 and IL-6 levels in WI-38 lung fibroblasts

**Figure 4:**
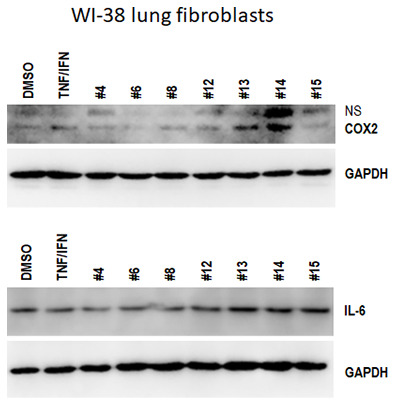
**Effects of novel *C. sativa* extracts on the levels of IL-6 and COX2 in WI-38 lung fibroblasts.** WI-38 cells grown to 80% confluency were treated with either 10 ng/ml TNFα /IFN γ alone or in combination with the indicated extracts; at 48 h after treatment, the whole cellular lysates were prepared and subjected to Western blot analysis using antibodies against IL-6 and COX2 as detailed in “Methods”. GAPDH served as a loading control.

While the observed effects were clearly interesting and intriguing, they called for more studies to analyze these effects in a lung model system. Thus, having seen promising effects of novel cannabis extracts on the levels of key inflammation modulators in EpiDermFT model we further proceeded to substantiate our data and analyze the effects of extracts on lung fibroblasts. WI-38 cells were exposed to either TNFα-IFNγ alone or in combination with the indicated extracts for 48 h, and Western blotting was performed to determine the effect on COX2 and IL-6 expression. We noted that COX2 was induced by TNFα-IFNγ, this induction was attenuated by extracts #4, #6, #8, #12, and #15, while enhanced by #13 and #14 (Figure 4). Albeit TNFα-IFNγ had no effect on IL-6 induction, extracts #4, #6, and #8 downregulated, while extracts #13, #14, and #15 upregulated the levels of IL-6 in WI-38 cells (Figure 4).

## DISCUSSION

Taken together, our results suggest that out of 7 studied extracts of novel *C. sativa* cultivars three were most effective down-regulating pro-inflammatory pathways and key cytokines implicated in the cytokine storm and ARDS in COVID-19. We noted that novel cannabis extracts down-regulated the levels of pro-inflammatory cytokine and interleukins, including IL-1 family, IL-23/IL-17 pathway, IL-6, IL-8, TNFα, and others that play key parts in inflammation and fibrosis. IL-1 family of interleukins is important in innate inflammation and autoimmunity [28]. IL-1α was shown to be constitutively present in numerous epithelial and mesenchymal cell types of healthy individuals, whereas IL-1β is mainly induced under disease conditions [28]. Both pro-inflammatory interleukins are upregulated in numerous inflammatory and autoinflammatory diseases and are important druggable targets. Recent studies show that levels of IL-1 were strongly elevated in individuals with COVID-19, and IL-1 levels correlated with disease severity [29]. Increased expression of IL-23/IL-17 pathway was previously correlated with pulmonary inflammation in polymicrobial sepsis [30]. While on the one hand, the IL-17 family confers protection from a variety of extracellular pathogens and was shown to drive leukocyte infiltration to facilitate clearance of infectious pathogens, aberrant IL-17 signaling can lead to excess inflammation and tissue damage and fibrosis [31], and has been implicated in ARDS, cystic fibrosis, and pulmonary fibrosis and other pathological conditions (reviewed in [31]).

Together with interleukins and TNFα genes, novel cannabis extracts regulated the expression of various other genes involved in fibrosis, including pulmonary fibrosis (PF) (Table 1). Among those were metalloproteinases (MMPs), key proteases involved in ECM remodeling [23]. MMP1, MMP2, MMP7, and MMP9 were previously reported to be upregulated in PF.

CCL2, also known as MCP-1 (Figure 1 and Table 1), which is an important hallmark of fibrosis, and has been indicated as a potential druggable anti-fibrotic target [23]. In previous studies, CCL2 was shown to promote fibroblast differentiation and facilitate their recruitment to the alveolar space, thus leading to excessive collagen deposition [32]. Besides, CCL2 promoted fibroblast survival and stimulated IL-6 production [33]. Importantly, along with CCL2, IL-1, IL-6 and TNFα also regulate fibrosis [23], and their down-regulation may be viewed as a potential anti-fibrotic effect.

Overall, extracts down-regulated many pro-fibrotic genes, such as WNT5A, iCAM and others. Previous studies have shown that *in vivo* inhibition of WNT-5A attenuated tissue destruction, improved lung function and restoration of alveolar epithelial cell markers expression in two animal models of COPD [24, 34]. Down-regulation of iCAM1 and iCAM5 genes is also an important finding, as the levels of iCAM1 were shown to be elevated in sera of PF patients [35], and recent studies showed that iCAM-1 inhibition reduced exacerbations of lung inflammation [36].

Interestingly, in our study, extracts #6 and #13, along with down-regulation of numerous pro-inflammatory cytokines, upregulated CXCL12. The role of CXCL12 upregulation in PF still needs to be fully established, but, based on the current knowledge, CXCL12 upregulation can be viewed as a potential PF contributor, and thus its upregulation may negate the potential benefits of cytokine down-regulation by extracts #6 and #13.

Overall, pronounced inhibition of TNFα and IL-6 is the most important finding, as these molecules are currently considered to be the key actionable targets in COVID-19 cytokine storm and ARDS. Anti-cytokine therapies are thought to be important for prevention of COVID-19 pneumonia [37], as currently there is a race to develop novel anti-cytokine storm regimens. To that effect several anti-cytokine therapies have been proposed and are now in clinical trials. These include anti-IL-6 receptor antibody tocilizumab [11, 12, 38], colchicine, an agent that can potentially influence levels of IL-6 and other cytokines [39], chloroquine [15], metronidazole [40], and statins [41], as well as melatonin as an anti-inflammatory adjuvant therapy [6]. Chloroquine has some immunomodulatory effects, potentially suppressing the production and release of TNF-α and IL-6 [15]. Colchicine has been shown to effectively suppress interleukin IL-1b, IL-18 and IL-6 in patients with acute coronary syndrome [42, 43] and is now being trialed in COVID-19 ARDS, albeit it also has very significant side effects [39]. Nonetheless, a lot of studies yielded negative or controversial results, calling for more efforts aimed at the discovery of novel anti-cytokine storm regimens.

Several rheumatological drugs are now being evaluated for therapeutic potential to tame COVID-19 pneumonia, ARDS, and prevent further complications such as PF [29]. Suppression of pro-inflammatory IL-1 family members and IL-6 has been shown to have a therapeutic effect in many inflammatory diseases, including viral infections, and has been explored as a potential therapeutic avenue in COVID-19 [44]. A recent review summarized the roles of IL-6 in COVID-19 pathogenesis and highlighted the important therapeutic potential of the IL-6 blockade in COVID-19 management [45]. Interestingly, a recent in-depth meta-analysis of 1302 COVID-19 cases showed that the level of IL-6 was 3-fold higher in patients with severe vs mild/moderate COVID-19 (*p* < 0.001). Furthermore, high IL-6 levels correlated with the development of severe lung damage (*p* = 0.001) [11].

Numerous reports based on several observational or non-placebo controlled studies in patients with severe COVID-19 and ARDS suggest the therapeutic potential of these agents, especially tocilizumab, a monoclonal antibody against IL-6 receptor (reviewed in [45]).

Tocilizumab, a humanized monoclonal antibody against the IL-6 receptor, is showing some promises, albeit it carries a hefty price tag and a lot of side effects [12, 46], and the results are not fully conclusive. Indeed, results of the COVIDOSE, low-dose tocilizumab in the treatment of Covid-19 trial, showed that low-dose tocilizumab administration led to rapid improvement in both laboratory and clinical manifestations of hyperinflammation in patients hospitalized with COVID-19 [47]. On the other hand, preliminary results of COVACTA clinical trial reported no statistical difference between tocilizumab vs placebo arm in severe COVID-19 [45]. Currently several clinical trials are ongoing to ascertain the efficacy of tocilizumab - NCT04445272, NCT04479358 (COVIDOSE-2), NCT04317092 (TOCIVID), NCT04345445, including a phase 3 RCT (NCT04412772).

In parallel, a study reported successful treatment of COVID-19 pneumonia with clazakizumab, monoclonal antibody against human IL-6 [48]. Currently, five RCTs are ongoing to ascertain the therapeutic potential of anti-IL-6 antibody in COVID-19 (NCT04381052, NCT04348500, NCT04494724, NCT04343989, NCT04343989).

TNFα not only is the main cytokine storm driver, it also was shown to mediate the transition from pulmonary inflammation to fibrosis [49]. As a pro-inflammatory cytokine, TNFα is mechanistically involved in lung and vascular tissue damage, ARDS and coagulopathy [50, 51]. Elevated levels of TNFα and other pro-inflammatory cytokines and chemokines, such as IL-6, IL-10 are risk factors for the development of severe COVID-19, and their levels are much higher in critical patients than in those with milder disease [50, 52]. Furthermore, most recent *in vitro* data show that TNFα facilitates SARS-CoV-2 interaction with ACE2 receptor that is a key gateway of the virus into human cells [53]. Moreover, recent clinical case reports have shown that the use of anti-TNF therapies in patients with rheumatological conditions and mild cases of COVID-19 prevented their further progression to the severe COVID-19 forms, most probably by mitigating deleterious effects of the high levels of TNFα and other cytokines that drive immunopathogenesis of COVID-19 [51]. Surprisingly, up to now, no TNFα inhibitors have been trialed for COVID-19. The expert commentary in Lancet stated that "trials of anti-tumour necrosis factor therapy for COVID-19 are urgently needed" [54]. To that effect, a recent expert review identified opportunities for the use of TNFα inhibitors in COVID-19 [29]. The first phase 2 study aimed to evaluate whether or not early administration of TNFα inhibition by infliximab in patients with severe COVID-19 will reduce disease duration and severity (NCT04425538).

While potentially effective, anti-TNFα and anti-IL-6 and other anti-inflammatory biologics are very expensive and cause an array of side effects, including malignancies and their efficacy needs to be ascertained in clinical trials. On the other hand, anti-TNFα and anti-IL-6 cannabis extracts that are generally regarded as safe (GRAS) modalities can be a useful addition to the current anti-inflammatory regimens to treat COVID-19, as well as various rheumatological diseases and conditions such as rheumatoid arthritis (RA), psoriasis and psoriatic arthritis, osteoarthritis, fibromyalgia, and others. Indeed, cultivars targeting TNFα, IL-6, IL-1β and causing concerted and significant downregulation of the rheumatoid arthritis pathway, pending thorough verification and clinical validation, may present a novel and promising natural resource for RA treatments and management of other TNFα, IL-6, IL-1β-mediated diseases. Furthermore, a recent report shows that CBD and the combination of major terpenes was far superior than dexamethasone in treating COVID-19 [55], albeit a full and final report of this study is still pending.

While potentially important, our study has limitations. It was initially based on the use of human EpiDermFT 3D tissue model which is not the closest to the lung tissues. That being said, inflammation can be effectively induced in this model and it was curbed by the application of extracts. Our data need to be further substantiated using more lung cell lines and 3D tissue models of inflammation. Importantly, extracts that curbed inflammation is 3D tissues also exerted anti-inflammatory potential in WI-38 lung fibroblasts, albeit not at the same level. In the future, it would be important to further expand the study and include analysis of the effects in lung 3D tissues. Furthermore, recent screen of the battery of high-CBD extracts identified several that inhibited COX2 and other inflammation makers in lung tissues (data not shown). Notwithstanding, our study laid a foundation for the future analysis of the anti-inflammatory potency of cannabis and its applications for COVID-19 and ARDS.

## CONCLUSIONS

Overall, we are the first to show that application of *C. sativa* extracts profoundly decreases the level of pro-inflammatory cytokines in human 3D tissues. Still, our study has several pitfalls. Here, we used human 3D full-thickness skin model to analyze the effects of cannabis extracts on inflammation and fibrosis. While it would be important to replicate the data in an airway epithelial and alveolar tissue models, and use either SARS-CoV2 virus or its components to induce inflammation, our data can be used as a roadmap for the future analysis. Moreover, key fundamental mechanisms of inflammation and fibrosis are similar in various tissues, and key roles of TNFα, IL-6 and other interleukins, chemokines, and MMPS have been well-established in an array of fibroproliferative diseases [5]. Pending further validation in lung tissue models, our novel extracts need to be studied in a clinical trial aimed to prevent or mitigate COVID-19 pneumonia and ARDS.

Most importantly, out of 7 selected extracts, only 3 performed best, one had no effects at all, and one exerted effects that may in turn appear to be deleterious, signifying that cannabis is not generic and careful cultivar selection must be based on thorough pre-clinical studies. Furthermore, the current study was developed to analyze the effects of medical cannabis applications rather than smoking.

In the future, anti-TNFα and anti-IL-6 extracts need to be analyzed for their potential to mitigate inflammation in rheumatoid arthritis, ankylosing spondylitis, and other rheumatologic conditions, especially given the fact that extracts profoundly downregulate the RA pathway and target TNFα and IL-6. Also, the effects of novel extracts also need to be analyzed for their potential to combat ‘inflammaging’ - the inflammatory underpinning of aging and frailty [56].

## MATERIALS AND METHODS

### Plant growth and extract preparation

All cannabis plants were grown in the licensed facility at the University of Lethbridge (license number LIC-62AHHG0R77-2019). *C. sativa* cultivars #4, #6, #8, #12, #13, #14, #15 were used for the experiments. Four plants per cultivar were grown at 22° C, 18 h light 6 h dark for 4 weeks and then transferred to the chambers with 12 h light/12 h dark regime to promote flowering. Plants were grown to maturity and flowers were harvested and dried. Flower samples from four plants per variety were combined and used for extraction. Three grams of the powdered plant tissue per each cultivar were used for extraction. Plant material was placed inside a 250 mL Erlenmeyer flask, 100 mL of ethyl acetate was poured into each flask. The flasks were covered with tin foil and incubated overnight in the dark at 21° C with continuous shaking at 120 rpm. Extracts were filtered, concentrated using a rotary vacuum evaporator and transferred to a tared 3-dram vial. The leftover solvent was evaporated to dryness in an oven overnight at 50° C to eliminate the solvent completely. Levels of cannabinoids was analysed using Agilent Technologies 1200 Series HPLC system. The extract stocks were prepared from the crude extracts, whereby 3-6 mg of crude extract was dissolved in DMSO (Dimethyl sulfoxide anhydrous, Life Technologies) to reach 60 mg/mL final concentration and stored at -20° C. Appropriate cell culture media (RPMI + 10% FBS or EMEM + 10% FBS) were used to dilute the 60 mg/mL stock to make working medium containing 0.01 mg/ml. Extracts were sterilized using 0.22 μm filter.

### Analysis of cannabinoids

Agilent Technologies 1200 Series HPLC system equipped with a G1315C DAD, G1316B column compartment, G1367D autosampler, and G1312B binary pump was used to analyse the acidic and neutral forms of phytocannabinoids. The separation was performed on a Phenomenex Kinetex EVO C18 column (5 μm, 100 x 2.1 mm id) with a Phenomenex SecurityGuard ULTRA guard column. Instrument control, data acquisition, and integration were done with ChemStation LC 3D Rev B.04.02 software (Agilent Technologies). A 2 μL injection volume was used for all calibration standards (THC, CBD, THC-A, CBD-A, CBG, CBG-A, all Sigma-Aldrich) and sample analysis. The compound peaks were detected for 230 nm and 280 nm. Mobile phases consisted of 50 mM ammonium formate (pH 5.19) (Sigma-Aldrich) in HPLC grade water (Fisher Chemical) on the A side and 100% methanol (Fisher Chemical) on the B side, with a flow rate 0.3 ml/min. Two samples per cultivar were analyzed, with two technical repeats per each sample. Data are presented in Table 3.

### Analysis of terpenes

Terpene analysis was performed on dry flowers of cultivars #6, #8 and #12 using a 8610C GC coupled with a flame ionization detector (FID) from SRI Instruments at Canvas Labs (Vancouver, BC, Canada). Two samples per cultivar were analyzed.

### Tissue and cell line models and treatments

### Tissue models

EpiDermFTTM tissues were purchased from Mattek Life Sciences (Ashland, MA), equilibrated overnight under standard culture conditions (37° C, 5% CO2) with EpiDermFT Assay Media (EFT-400-ASY) and cultured according to manufacturer’s instructions. Three tissues were used per extract. EpiDermFT recreates normal skin tissue structure with differentiated dermis and epidermis. It consists of human-derived epidermal keratinocytes and dermal fibroblasts that are mitotically and metabolically active. The tissues were cultured according to the manufacturer's protocol, using an air-liquid interface tissue culture technique.

To induce inflammation, tissues were exposed to UVC for 2 min, receiving 7000 erg. Distance from the light source was set to 10 cm. Upon exposure, tissues were treated with extracts. Specifically, right after the UVC treatment, the cannabis extracts (15 μl per sample) or vehicle (DMSO) were dissolved in media and applied to the media surrounding the tissues (n=3 for each condition). Control samples (PBS and DMSO) were sham treated – carried to the UVC source etc. but no UVC was given. The following experimental groups were set up:

“UNT” – untreated tissues;

“PBS” - 15 μl of 30% glycerol in PBS was applied to the tissues and no exposure was done;

“DMSO” - 15 μl of DMSO (0.017% in 30% glycerol-PBS) was applied to the tissues and no exposure was done;

“UV-PBS” - tissues were exposed to UV and 15 μl of 30% glycerol-PBS was applied to them;

“UV-DMSO” – tissues were exposed to UV and 15 μl of DMSO (0.017% in 30% glycerol-PBS) was applied to them;

“#4” through “#15” - tissues were exposed to UV and 15 μl of extracts in DMSO was applied to them.

Tissues were incubated with extracts for 24 h and flash frozen for RNA and protein analysis.

### Cell line

WI-38 lung fibroblasts were purchased from the ATCC and cultured in Eagle’s Minimum Essential Medium (EMEM) supplemented with 10 fetal bovine serum according to the manufacturer’s instructions. WI-38 cells grown to 80% confluency were treated with proinflammatory cytokines (10 ng/ml TNFα /IFN γ) alone or in combination with 0.015 μb/μL extracts, vehicle (DMSO) served as a control. At 48 h after treatment, the cells were washed twice with ice-cold PBS and lysed in a radioimmunoprecipitation assay buffer (RIPA).

### Gene expression analysis

### RNA isolation

Three tissues per group were used for the analysis of gene expression profiles. RNA was isolated from tissues using TRIzol® Reagent (Invitrogen, Carlsbad, CA), further purified using an RNAesy kit (Qiagen), and quantified using Nanodrop2000c (ThermoScientific). Afterwards, RNA integrity and concentration were determined using 2100 BioAnalyzer (Agilent).

### Library construction and sequencing

In all cases, the sequencing libraries were prepared using NEBNext Ultra II mRNA library preparation kit for Illumina (NEB) following the manufacturer’s instructions. The samples were processed by the same technician at the same time to avoid the introduction of technical batch effects. The cDNA fragment libraries were sequenced using NextSeq500 sequencing analyzer (Illumina). The samples were balanced evenly across the lanes of the sequencing flowcell.

### Bioinformatics analysis

Base-calling and demultiplexing were done with Illumina CASAVA v.1.9 bioinformatics pipeline. The base qualities were examined using FastQC v.0.11.8. The adapters and low-quality bases were trimmed using TrimGalore!v.0.6.4 https://www.bioinformatics.babraham.ac.uk/projects/trim_galore/. Trimmed reads were mapped to the human genome version GRCh37 using HISAT2 version 2.0.5 [57]. Counts of reads mapping to the gene as a meta-feature were obtained using featureCounts v.1.6.1 [58] taking to account the directionality of the sequencing libraries. Counts of reads mapping to features were loaded into R v.3.6.1 and normalized using DESeq2 v.1.24.0 Bioconductor package as described in the manual [59]. The differences between all experimental groups were examined using the likelihood ratio test (LRT) test implemented in DESeq2. The reduced model included the intercept and the full model was the experimental group (Cannabis extracts and controls).

Pathway visualization was conducted using pathview v.1.26.0 Bioconductor package based on pathway schemes downloaded from Kyoto Encyclopedia of Genes and Genomes (KEGG) [60, 61]. Generally applicable gene set enrichment (GAGE) for pathway analysis method was used in unidirectional mode to detect experimentally perturbed KEGG pathways [62].

### Statistics

Multiple comparisons adjustment of p-values was done using Benjamini-Hochberg procedure [63]. Specific comparisons between groups were extracted using *results()* function with *contrast* argument specified. Genes with adjusted p-values below 0.05 were considered significant.

### Western blot analysis

After treatment with cannabis extracts for the indicated time, whole cellular lysates of 3D tissues were prepared in radioimmunoprecipitation assay buffer using 2.0 mm ZR BashingBead beads (Zymo Research). Proteins (30-100 μg per sample) were electrophoresed in 10% sodium dodecyl sulfate polyacrylamide gel and electrophoretically transferred to polyvinylidene difluoride membranes (Amersham Hybond^TM^-P, GE Healthcare) at 4° C for 1.5 h. The blots were incubated for 1 h with 5% nonfat dry milk to block nonspecific binding sites and subsequently incubated at 4° C overnight with 1:1000 dilution of polyclonal antibody against IL-6 and COX-2 (Abcam). Immunoreactivity was detected using a peroxidase-conjugated antibody and visualized with the ECL Plus Western Blotting Detection System (GE Healthcare). The blots were stripped before reprobing with antibody against actin (Santa Cruz Biotechnology) or GAPDH (Abcam).

## Supplementary Material

Supplementary Figure 1
