# Supplementary material for: Fighting the storm: could novel anti-TNFα and anti-IL-6 C. sativa cultivars tame cytokine storm in COVID-19?
Source: Aging (Albany NY). 2021 Jan 19;13(2):1571–90. doi: 10.18632/aging.202500 (PMC7880317; doi:10.18632/aging.202500)
Supplement: Supplementary Figure 1 [file aging-13-202500-s001.pdf]

SUPPLEMENTARY FIGURE

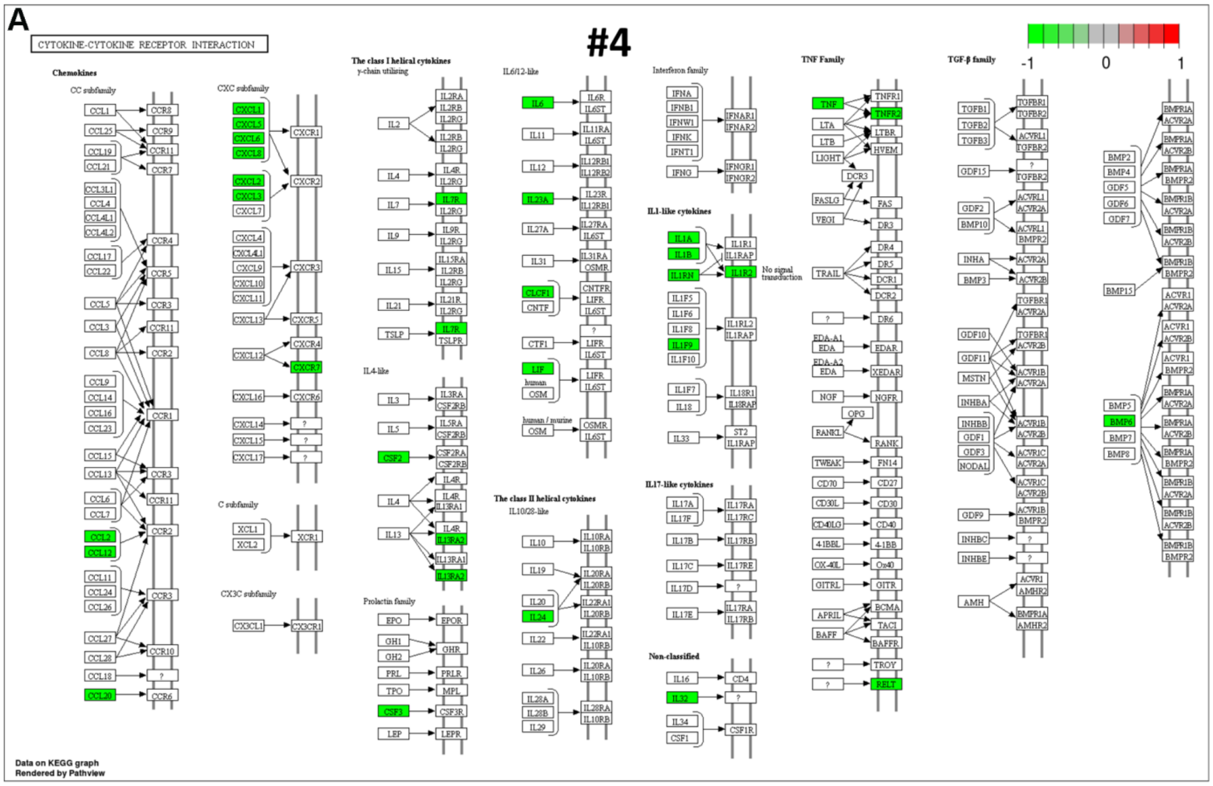

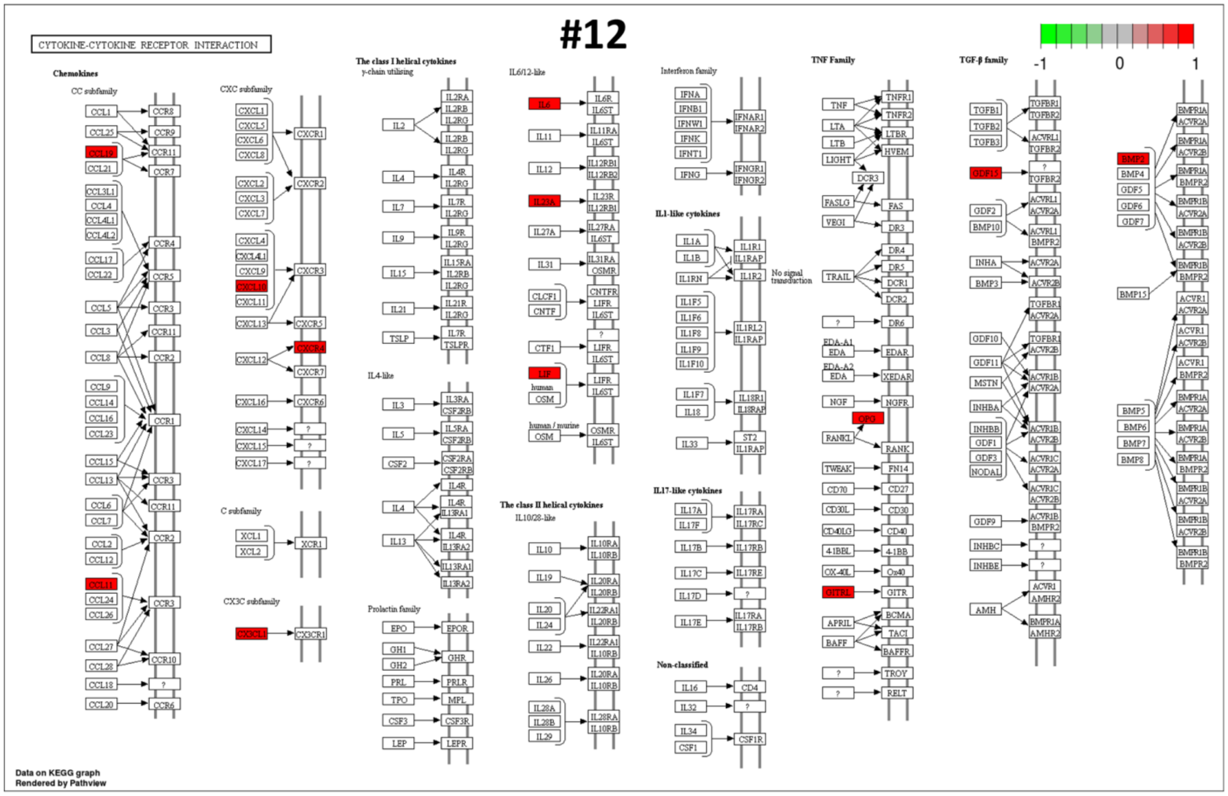

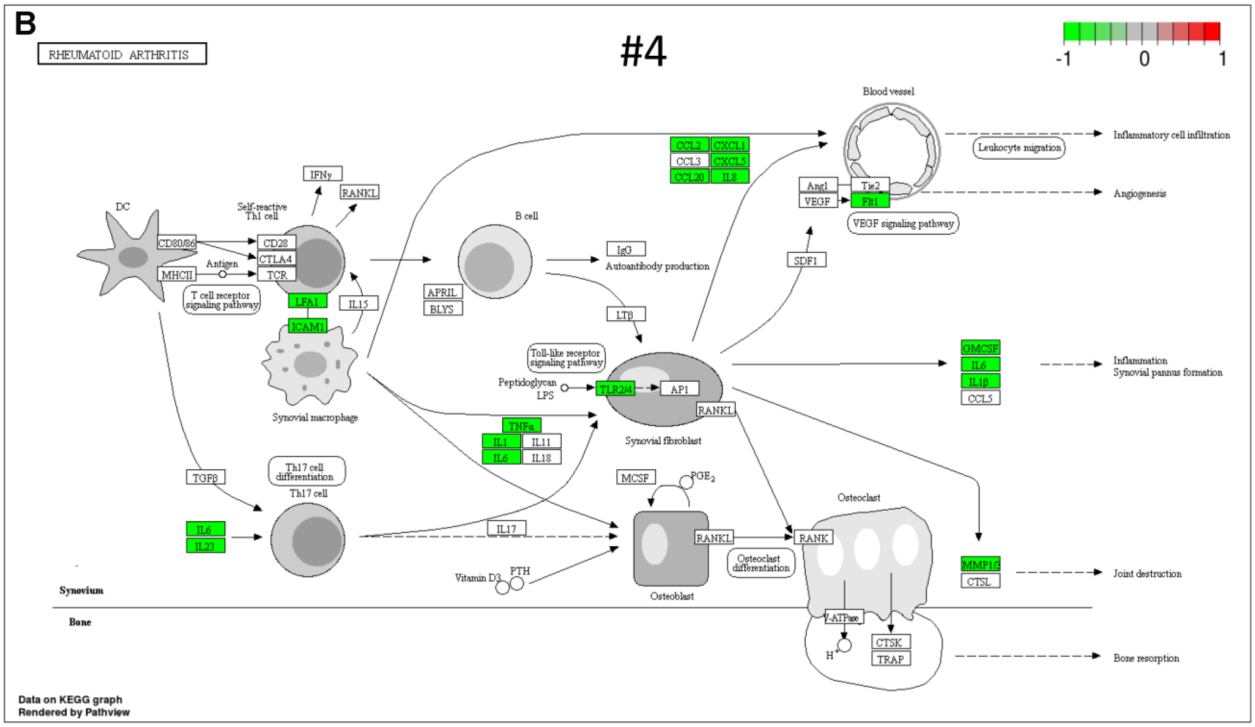
B
RHEUMATOID ARTHRITIS
#4
-1
0
1
Blood vessel
Inflammatory cell infiltration
Leukocyte migration
Angiogenesis
CCL2
CXCL1
CCL3
CXCL5
CCL20
IL8
Ang1
Tie2
VEGF
Flt1
VEGF signaling pathway
IFNγ
RANKL
DC
Self-reactive Th1 cell
B cell
CD80/86
CD28
CTLA4
Antigen
MHCII
TCR
T cell receptor signaling pathway
LFA1
ICAM1
IL15
APRIL
BLYS
IgG
Autoantibody production
LTβ
SDF1
Toll-like receptor signaling pathway
Peptidoglycan
LPS
TLR2/4
AP1
RANKL
GMCSF
IL6
IL1β
CCL5
Inflammation
Synovial pannus formation
Synovial macrophage
TNFα
IL1
IL11
IL6
IL18
Synovial fibroblast
TGFβ
Th17 cell differentiation
Th17 cell
MCSF
PGE2
IL6
IL23
IL17
RANKL
RANK
Osteoclast
Osteoclast differentiation
MMP1/3
CTSL
Joint destruction
Vitamin D3
PTH
Osteoblast
Synovium
Bone
V-ATPase
H+
CTSK
TRAP
Bone resorption
Data on KEGG graph
Rendered by Pathview

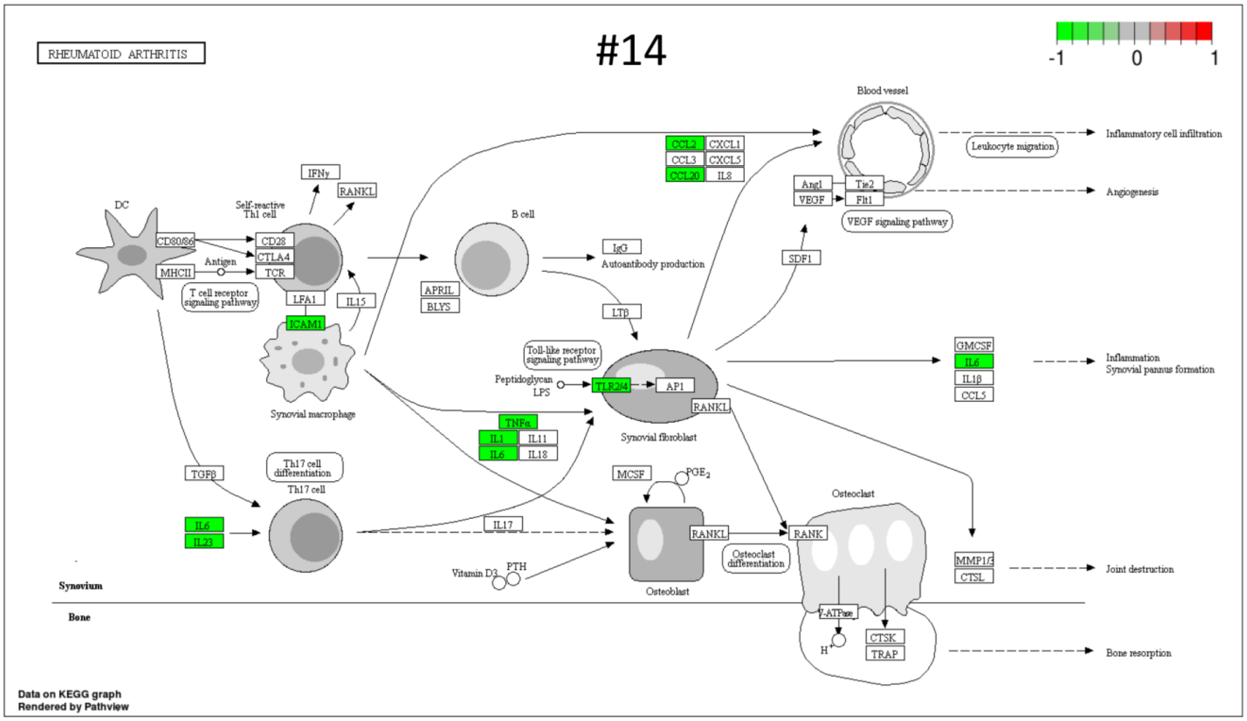
RHEUMATOID ARTHRITIS
#14
-1
0
1
Blood vessel
Inflammatory cell infiltration
Leukocyte migration
Angiogenesis
CCL2
CXCL1
CCL3
CXCL5
CCL20
IL8
Ang1
Tie2
VEGF
Flt1
VEGF signaling pathway
IFNγ
RANKL
DC
Self-reactive Th1 cell
B cell
CD80/86
CD28
CTLA4
Antigen
MHCII
TCR
T cell receptor signaling pathway
LFA1
ICAM1
IL15
APRIL
BLYS
IgG
Autoantibody production
LTβ
SDF1
Toll-like receptor signaling pathway
Peptidoglycan
LPS
TLR2/4
AP1
RANKL
GMCSF
IL6
IL1β
CCL5
Inflammation
Synovial pannus formation
Synovial macrophage
TNFα
IL1
IL11
IL6
IL18
Synovial fibroblast
TGFβ
Th17 cell differentiation
Th17 cell
MCSF
PGE2
IL6
IL23
IL17
RANKL
RANK
Osteoclast
Osteoclast differentiation
MMP1/3
CTSL
Joint destruction
Vitamin D3
PTH
Osteoblast
Synovium
Bone
V-ATPase
H+
CTSK
TRAP
Bone resorption
Data on KEGG graph
Rendered by Pathview

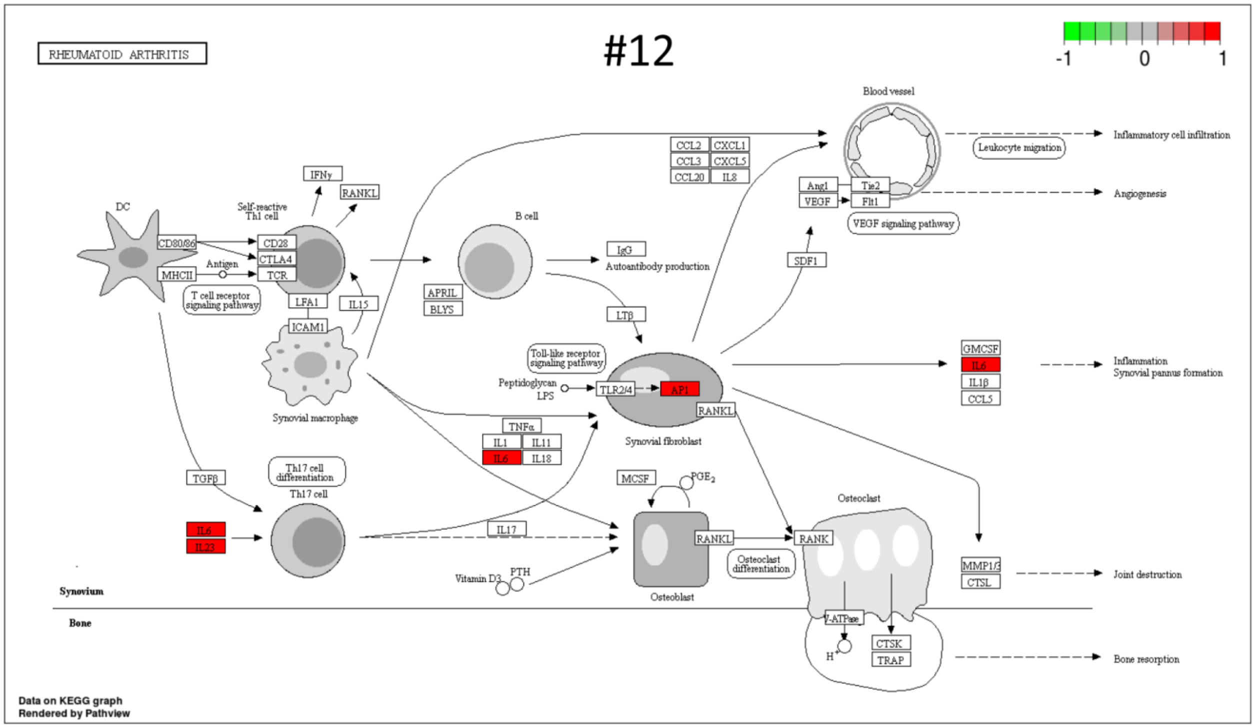

Supplementary Figure 1. Effects of selected extracts on 3D tissues on cytokine-cytokine receptor interactions and rheumatoid arthritis pathways. Generally applicable gene set enrichment (GAGE) for pathway analysis method was used in unidirectional mode to detect experimentally perturbed KEGG pathways [62]. (**A**) Changes in the cytokine-cytokine receptor interactions pathway caused by extracts #4 and #12. (**B**) Changes in the rheumatoid arthritis pathway caused by extracts #4, #14 and #12. Red - upregulation; green – down-regulation.
